# Downregulation of protein kinase C gamma reduces epithelial property and enhances malignant phenotypes in colorectal cancer cells

**DOI:** 10.1016/j.isci.2022.105501

**Published:** 2022-11-04

**Authors:** Reiko Satow, Yudai Suzuki, Shinobu Asada, Sae Ota, Masashi Idogawa, Shiori Kubota, Noi Ikeo, Atsuko Yoneda, Kiyoko Fukami

**Affiliations:** 1 Laboratory of Genome and Biosignals, Tokyo University of Pharmacy and Life Sciences, Hachioji-shi, Tokyo 192-0392, Japan; 2 Department of Medical Genome Sciences, Research Institute for Frontier Medicine, Sapporo Medical University School of Medicine, Sapporo, Japan

**Keywords:** Molecular biology, Cancer

## Abstract

Loss of epithelial integrity is associated with colorectal cancer (CRC) aggressiveness. Protein kinase C (PKC) is frequently implicated in human cancers, but the role of PKCγ in CRC remains poorly understood. Here, we show that PKCγ, a conventional PKC, is expressed in normal colonic epithelium, but this is lower in dedifferentiated CRC. PKCγ expression was downregulated by SNAI1 overexpression, and low PKCγ expression was associated with poor prognosis in patients with CRC. Transient or stable knockdown of PKCγ reduced E-cadherin expression in CRC cells. PKCγ knockdown enhanced proliferation, anchorage-independent cell growth, resistance to anti-cancer drugs, and *in vivo* tumor growth of DLD-1 cells. We have also identified phosphorylation substrates for PKCγ. Among them, ARHGEF18, a RhoA activator that stabilizes cell-cell junctions, was phosphorylated and stabilized by PKCγ. Thus, these results suggest that the downregulation of PKCγ decreases the epithelial property of CRC cells and enhances its malignant phenotypes.

## Highlights

• Low PKCγ expression is associated with a poor prognosis in patients with CRC
• The expression of PKCγ is associated with the epithelial properties of CRC cells
• PKCγ knockdown induces malignant phenotypes in CRC cells
• PKCγ phosphorylates and stabilizes ARHGEF18, that stabilizes cell-cell junctions

## Graphical abstract

**Figure:**
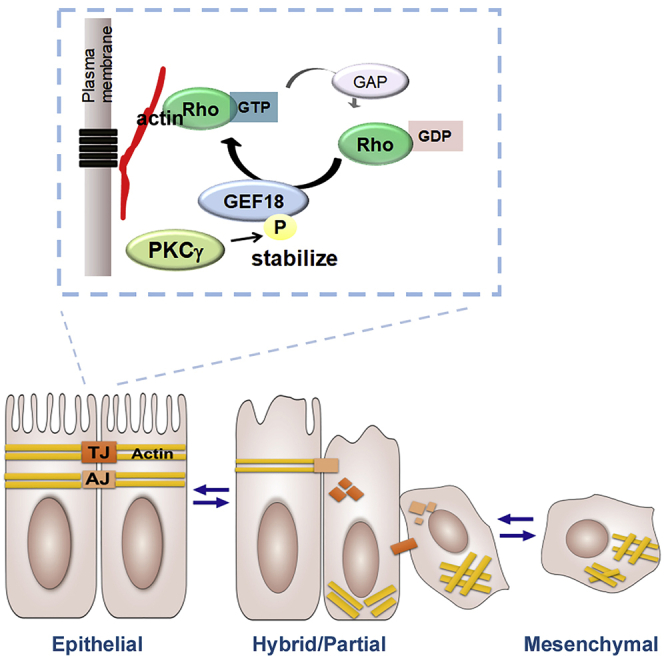

## Introduction

Although several drugs targeting specific molecules have been developed and are used clinically for colorectal cancer (CRC) treatment, the clinical outcome of patients with CRC with metastasis is still poor.^1^ Therefore, further research is required to determine the molecular mechanisms of CRC progression to develop more effective drugs. Cancer metastasis is often driven by the epithelial–mesenchymal transition (EMT), which is a crucial cellular program that enables epithelial cells to acquire an invasive phenotype for metastatic progression.^2^ EMT permits epithelial cells to acquire a mesenchymal morphology accompanied by the loss of the apical–basal polarity and disassembly of epithelial cell–cell contacts including tight junctions and adherens junctions. Recent studies found that migrating cancer cells frequently display a partial or transient EMT state in which various combinations of epithelial and mesenchymal properties coexist.^3^

E-cadherin is a significant mediator of cell-cell adhesion in epithelial tissue, and loss of E-cadherin is a critical step in the loss of epithelial property. In CRC cells, loss of E-cadherin is a hallmark of EMT, and E-cadherin is a modulator of cell biological traits, as the depletion of E-cadherin by small interfering RNA (siRNA) promotes cell growth, invasion, and drug resistance through the induction of β-catenin nuclear translocation.^4^ In addition, the loss of cell–cell contact promotes β-catenin signaling, which then activates EMT-inducing transcription factor to repress E-cadherin transcription.^5^ Thus, E-cadherin loss and EMT are mutually related.

EMT-inducing transcription factors such as SNAI1 (also known as SNAIL), SNAI2 (also known as SLUG), ZEB1, and TWIST repress the genes associated with the epithelial phenotype. SNAI1 binds to E-box sequences in the promoter region of *CDH1* (encoding E-cadherin) and recruits the polycomb repressive complex to repress transcription.^6^^,^^7^^,^^8^ SNAIL also represses the expression of genes regulating tight junction and apical–basal polarity.^9^ In CRC, SNAIL induces features of cancer stem cells, including chemoresistance, radioresistance, and the ability to initiate tumor formation.^10^ EMT also enhances cancer stemness and resistance to therapeutic agents.^2^

Previously, we have shown that phospholipase C delta 1 (PLCδ1) contributes to E-cadherin expression to suppress CRC aggressiveness.^11^ During our attempt to identify PKC isoforms that can be regulated by PLCδ1 in CRC, we noticed the novel role of PKCγ in CRC cells. PKCs are serine/threonine kinases that can be classified into three groups: “conventional” (cPKCs), “novel” (nPKCs), and “atypical” (aPKCs). Of these three groups, only cPKCs are activated by both calcium ions and DAG. The cPKCs comprise PKCα (*PRKCA*), two splice variants of PKCβ (*PRKCB*), and PKCγ (*PRKCG*).^12^ Some previous reports have suggested tumor-suppressive roles for PKCα and PKCβ,^13^^,^^14^ however, the role of PKCγ in CRC is not completely characterized. In this study, we have characterized the roles of PKCγ in CRC.

## Results

### The expression of PKCγ is associated with the epithelial properties of colorectal cancer cells

**Figure 1:**
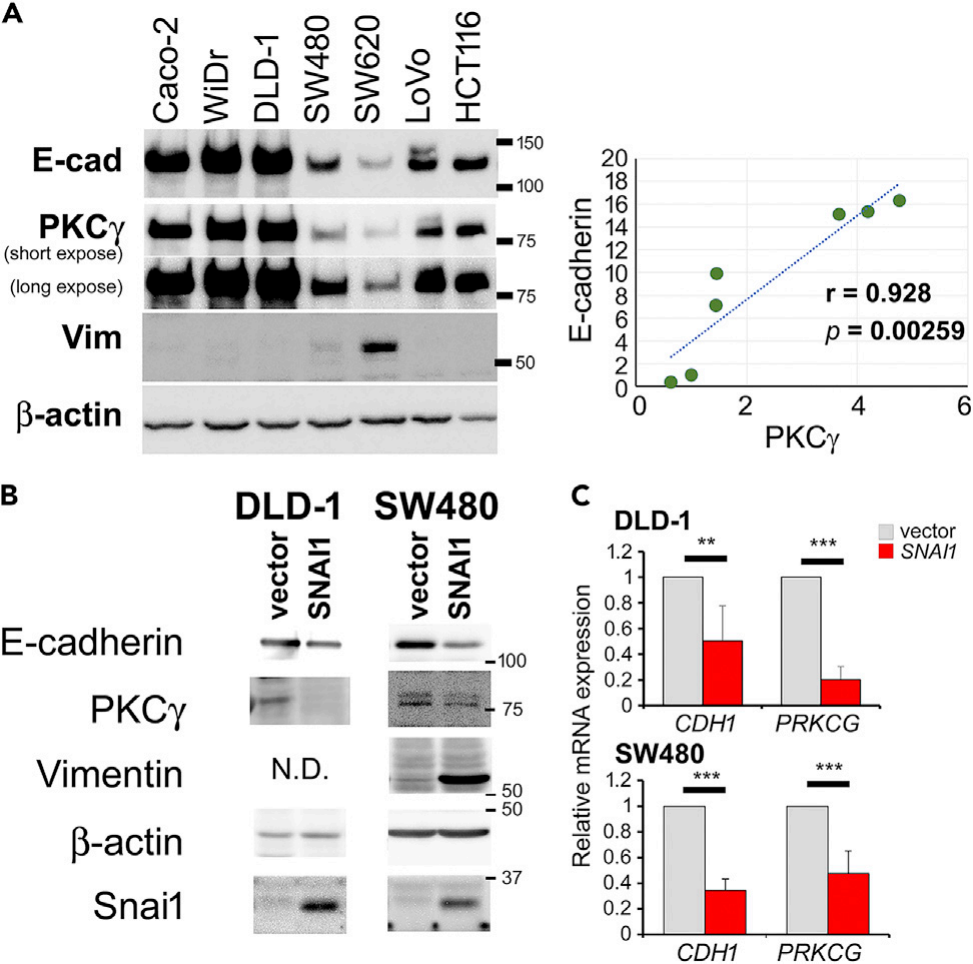
PKCγ expression is associated with the epithelial properties of CRC cells (A) The expression of PKCγ and E-cadherin in CaCo-2, WiDr, DLD-1, SW480, SW620, Lovo, and HCT116 cells was quantified using western blotting. The correlation between PKCγ and E-cadherin expression was assessed using Pearson’s product-moment correlation test. (B) Western blot analyses of PKCγ and other marker proteins in DLD-1 and SW480 cells expressing SNAI1. (C) The relative expression levels of *CDH1* (E-cadherin) and *PRKCG* (PKCγ) normalized to that of *GAPDH*, were determined by qPCR analyses in DLD-1 and SW480 cells expressing SNAI1 (n = 6, each). Data represent means ± SD. Statistical analysis was performed using Student’s *t* test. ∗∗p < 0.01; ∗∗∗p < 0.001. See also Figures S1 and S2, and Table S1.

To assess the expression of PKCγ, western blot analysis of the CRC cell lines Caco-2, WiDr, DLD-1, SW480, SW620, Lovo, and HCT116 was performed. The specificity of the PKC antibodies used in this study was confirmed (Figure S1). The characteristics of the CRC cell lines used in this study are summarized in Table S1. Among these cell lines, only SW620 showed pronounced expression of the mesenchymal marker, vimentin. The expression of both PKCγ and E-cadherin was the lowest in the SW620 cells (Figure 1A). The other cell lines showed little or no vimentin expression, suggesting that they did not undergo EMT, although the possibility of partial EMT cannot be excluded. The results revealed that the amount of PKCγ and E-cadherin proteins in these cells were significantly correlated (r = 0.928, *p* = 0.00259) (Figure 1A).

To explore the relationship between epithelial state and PKCγ expression, DLD-1 and SW480 cells (used mainly because both these cell lines have mutations in both *KRAS* and *TP53*, which are frequently observed in CRC, and they retain epithelial traits) were stably overexpressed with SNAI1, an EMT-inducible transcription factor associated with CRC.^15^ SNAI1 induced EMT, as demonstrated by a reduction in E-cadherin and an upregulation of vimentin expression (Figures 1B and 1C). SNAI1 transcriptionally repressed PKCγ (Figure 1C). Furthermore, six putative SNAI1-binding sites (E-box; CANNTG) were identified in the proximal promoter region of *PRKCG* (encoding PKCγ). To assess whether SNAI1 regulates *PRKCG* expression via the E-box region, we performed reporter assays using reporter constructs containing the *PRKCG* proximal promoter region. SNAI1 downregulated *PRKCG* promoter activity (Figure S2A), and mutagenesis in each E-box (CANNTG to ATNNTG) revealed two E-boxes (E1 and E3) that were essential for the suppression (Figure S2B). Because loss of epithelial property enhances additional EMT transcriptional factors, such as SNAI2, which also binds to the E-box, SNAI1 could regulate *PRKCG* directly and/or indirectly.^5^^,^^16^ These results suggest that PKCγ expression is associated with the epithelial property of CRC.

### PKCγ expression is suppressed in dedifferentiated colorectal cancers

**Figure 2:**
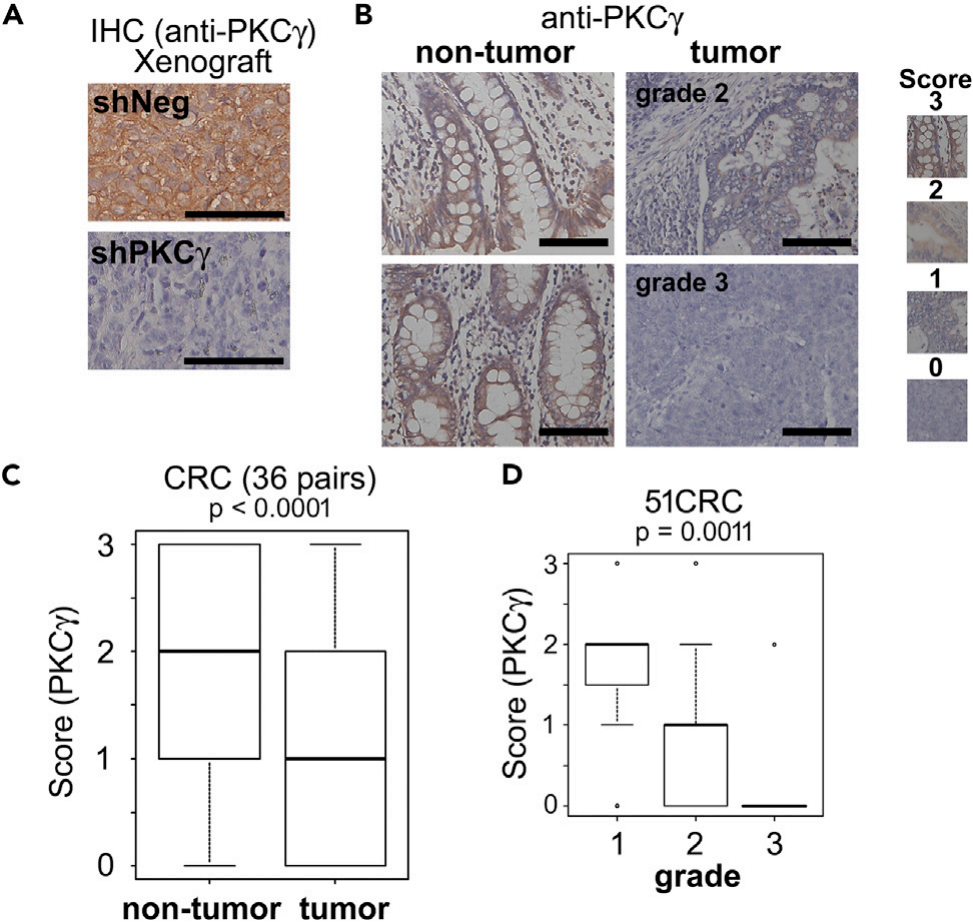
PKCγ expression is lower in dedifferentiated CRCs (A) Positive control of immunohistochemical study using xenografts of DLD-1 cells suppressing PKCγ expression using the anti-PKCγ antibody. Scale bar: 100 μm. (B–D) A human CRC tissue array, which contains 36 pairs of non-tumor colon epithelium and CRC tissues, and 15 other CRC samples, was probed with the anti-PKCγ antibody. (B) Representative images of grade 2 (moderately differentiated) or grade 3 (poorly differentiated) tumors and paired non-tumor samples are shown. Scale bar: 100 μm. (C) The PKCγ expression levels were assessed using the Wilcoxon signed-rank test (36 pairs). (D) The relationship between the expression of PKCγ and the tumor grade in 51 CRC samples was assessed using Fisher exact test. See also Table S2.

Using human CRC tissue microarrays, we next analyzed the expression of PKCγ in clinical samples. Immunohistochemical staining with anti-PKCγ antibody (positive control staining is shown in Figure 2A) revealed that PKCγ is expressed in normal colonic epithelium and that its expression is lower in CRCs, especially in dedifferentiated CRC (Figures 2B–2D, and Table S2).

### PKCγ knockdown reduces E-cadherin expression and induces malignant phenotypes in colorectal cancer cells

**Figure 3:**
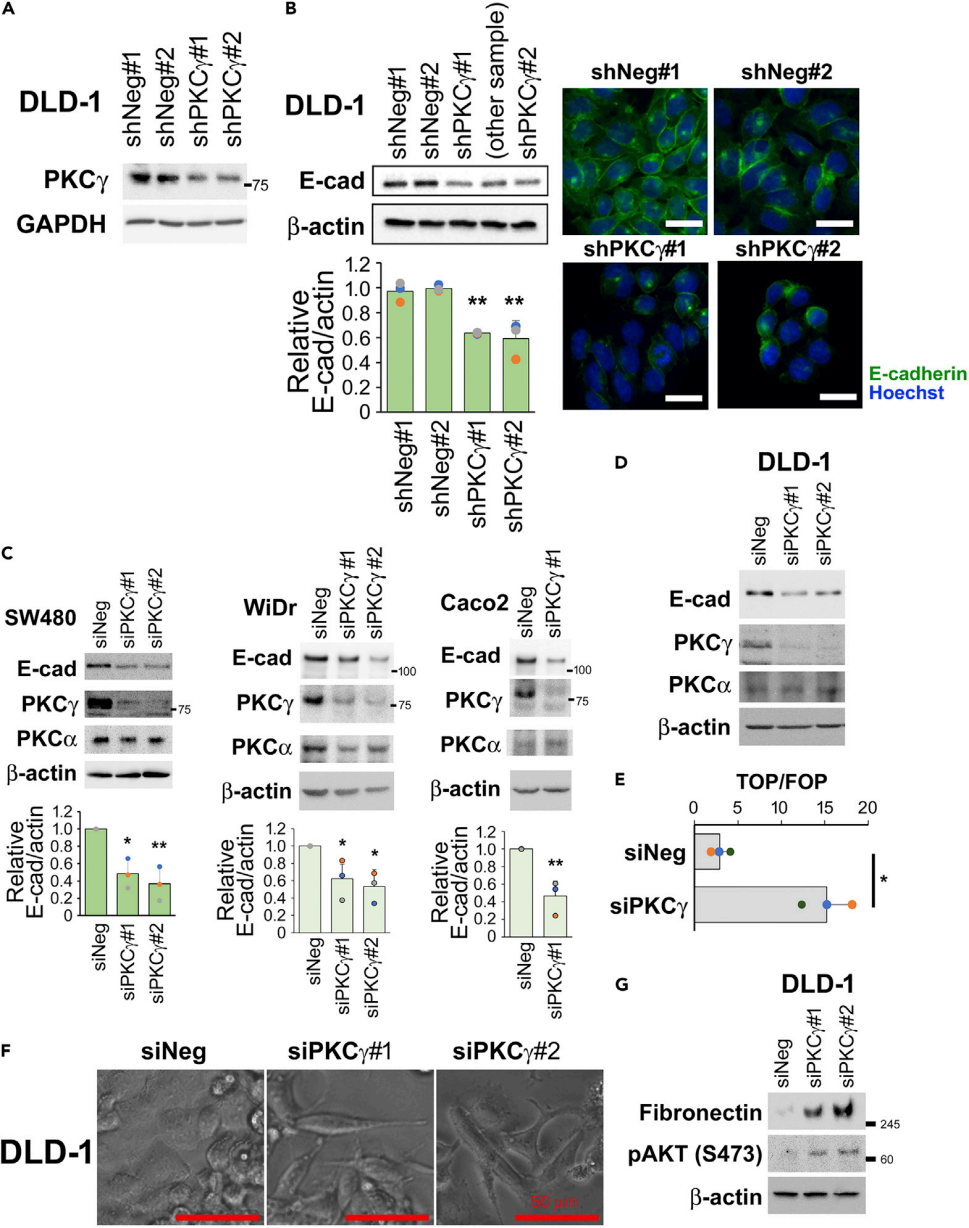
PKCγ knockdown reduces E-cadherin expression in CRC cells (A) DLD-1 cells suppressing PKCγ (shPKCγ#1, #2) or negative control clones (shNeg#1, #2) were assessed by western blotting. (B) DLD-1 cells transfected with shPKCγ (shPKCγ#1, #2) or negative control clones (shNeg#1, #2) were assessed by western blotting for E-cadherin. Immunofluorescence staining of E-cadherin (green) with an anti-E-cadherin antibody and counter nuclear staining using Hoechst (blue) is shown. The bar indicates 25 μm. (C) SW480, WiDr, and Caco-2 cells were transfected with siPKCγ#1 and #2 and assessed using western blotting with the indicated antibodies (n = 3). (D) DLD-1 cells were transfected with siRNA for PKCγ, and after 6 days, the cells were assessed by western blotting using the indicated antibodies. (E) DLD-1 cells were transfected with siPKCγ#1 and assessed for the TOP/FOP FLASH assay. (F) Phase contrast images of DLD-1 cells transected with indicated siRNA and cultured on collagen-coated plates. The bar indicates 50 μm. (G) Western blot analysis of DLD-1 cells transfected with the indicated siRNA, cultured on collagen-coated plates with 10% serum-containing medium for 5 days, and then cultured in serum-free medium for 24 h. (B, C, and E) Data represent means ± SD. Statistical analysis was performed using Dunnett’s multiple comparison of means test (B and C) or Student’s *t* test (E). ∗p < 0.05; ∗∗p < 0.01. See also Figure S3.

To investigate the role of PKCγ in CRC, we stably knocked down *PKCγ* in the CRC cell line DLD-1 (Figure 3A). The knockdown of PKCγ reduced E-cadherin expression (Figure 3B). Transient knocked down of *PKCγ* in CRC cell lines, SW480, WiDr, Caco-2, and DLD-1 also caused E-cadherin reduction (Figures 3C and 3D). Furthermore, because PKC isoforms can affect each other,^14^ the expression of PKCα, which is expressed in the colon epithelium, was also assessed (Figures 3C and 3D). In some cases, PKCα expression was partially reduced by transfection with PKC*γ* siRNA (Figure 3, WiDr). However, the knockdown of PKCα in these cells did not cause a reduction in E-cadherin (Figure S3). Thus, PKCγ, but not PKCα, is responsible for E-cadherin expression.

**Figure 4:**
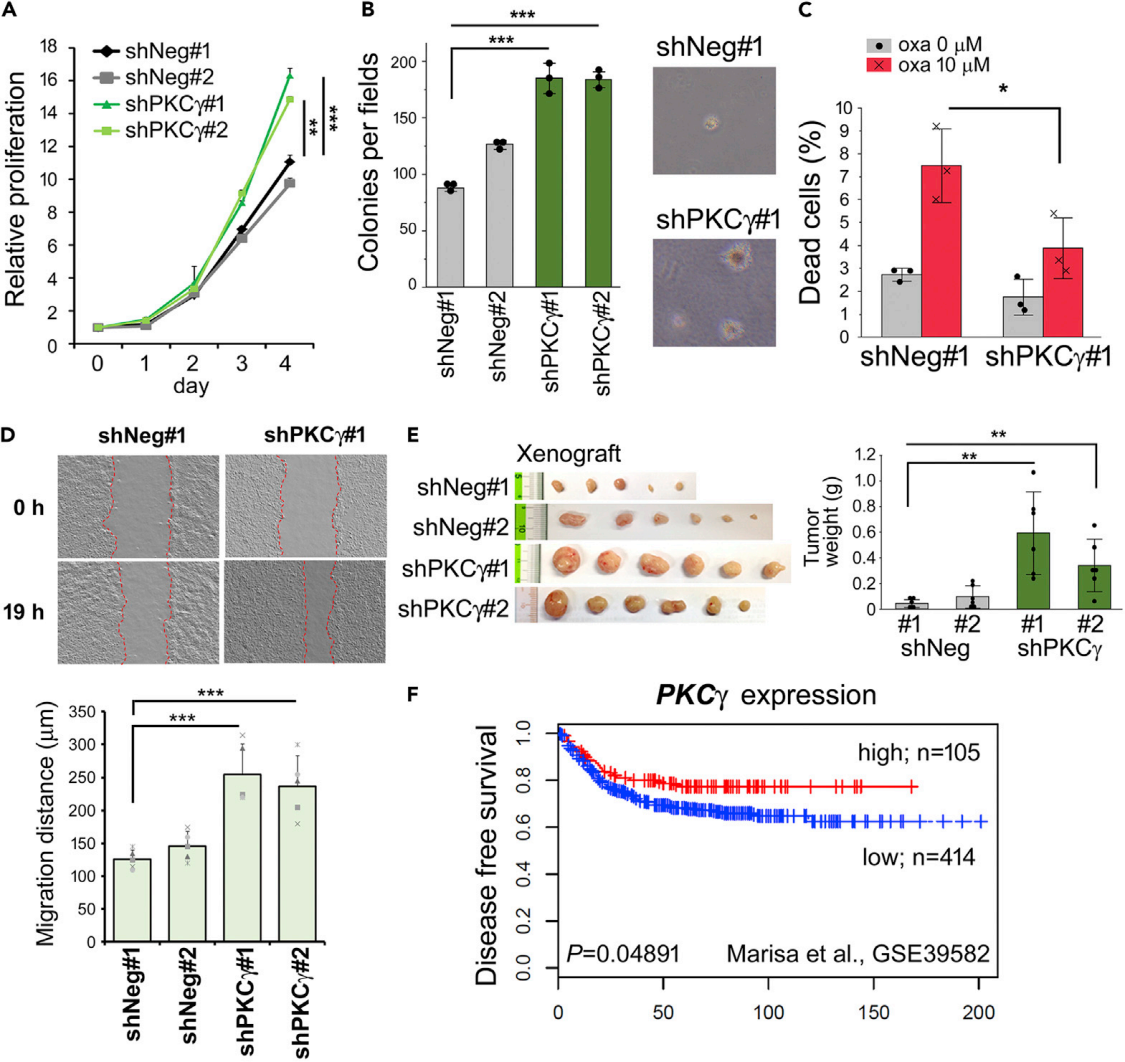
PKCγ knockdown enhances malignant phenotypes in CRC cells (A) The cell number was determined at the indicated times and the relative proliferation is shown (n = 3). (B) Cells were seeded in 6-well plates with soft agar and after 2–3 weeks, the numbers of colonies were counted (n = 3). Representative images of the colonies are also shown. (C) Cells were treated with 10 μM oxaliplatin (oxa) for 48 h and then incubated with FITC-labeled annexin V, and the percentage of annexin V-positive cells was determined (n = 3). (D) Confluent cells were starved for 24 h and the cell layers were scratched. Images at the same position were obtained before and after 19 h incubation. (E) Cells were inoculated into the flanks of nude mice. Representative images of the xenografts 28 days after inoculation are shown in the upper panels, when the xenografts were weighed (shNeg#1; n = 5. shNeg#2, shPKCg#1, #2; n = 6, each). Data represent means ± s.e.. (F) The relationship between *PKCγ* expression (GSE39582; Affymetrix microarray probe:206,270_at) and survival was determined and plotted using the Kaplan–Meier method. The disease-free survival rate for patients with high or low *PKCγ* expression is plotted as red and blue lines, respectively. (A–D) Data represent means ± SD. Statistical analysis was performed using Dunnett’s multiple comparison of means test (A, B, and D), or Student’s *t* test (C). ∗p < 0.05; ∗∗p < 0.01; ∗∗∗p < 0.001.

Because a reduction in E-cadherin promotes morphological change, cell growth at high cell density, drug resistance, and survival through the induction of β-catenin nuclear translocation,^4^^,^^17^ we next assessed β-catenin-TCF/LEF-mediated transcription reporter assay (TOP/FOP FLASH). Knockdown of PKCγ in DLD-1 cells led to significant activation of TOP FLASH activity (Figure 3E). Indeed, the knockdown of PKCγ induced mesenchymal morphology to DLD-1 cells (Figure 3F). Mesenchymal marker fibronectin and the phosphorylation of related survival factor AKT^18^^,^^19^^,^^20^ were markedly upregulated upon PKCγ knockdown (Figure 3G). However, vimentin expression was not induced in these cells (data not shown), suggesting knockdown of PKCγ-induced partial EMT. Knockdown of PKCγ had a minimal effect on cell proliferation by 48 h, but the proliferation rate of PKCγ-suppressed cells increased after 3–4 days (Figure 4A). Soft agar assays revealed that PKCγ knockdown enhanced the anchorage-independent growth of DLD-1 cells (Figure 4B). Furthermore, PKCγ*-*suppressed cells showed less oxaliplatin-induced cell death, suggesting that PKCγ knockdown also enhances the resistance to oxaliplatin in DLD-1 cells (Figure 4C). Scratch migration assay revealed that PKCγ*-*knockdown enhanced the migration of DLD-1 cells (Figure 4D). We next performed *in vivo* experiments to evaluate the roles of PKCγ in tumor malignancy. Xenograft assays showed that PKCγ knockdown significantly enhances *in vivo* tumor growth of DLD-1 cells (Figure 4E). These results indicate that PKCγ has suppressive roles in CRC cell proliferation, anchorage-independent cell growth, resistance to anti-cancer drugs, migration, and *in vivo* tumor growth.

### Low PKCγ expression is associated with poor prognosis in patients with colorectal cancer

To determine whether *PKCγ* expression affects prognosis, we examined gene expression datasets from patients with CRC,^21^ and constructed survival curves using the Kaplan–Meier method. In colon cancer datasets, the incidence of disease-free survival was significantly lower in patients whose tumors had low *PKCγ* expression than in those who had high *PKCγ-*expressing tumors (Figure 4F). These results support the idea that *PKCγ* has suppressive roles in CRC progression.

### Identification of the phosphorylation substrates of PKCγ

**Figure 5:**
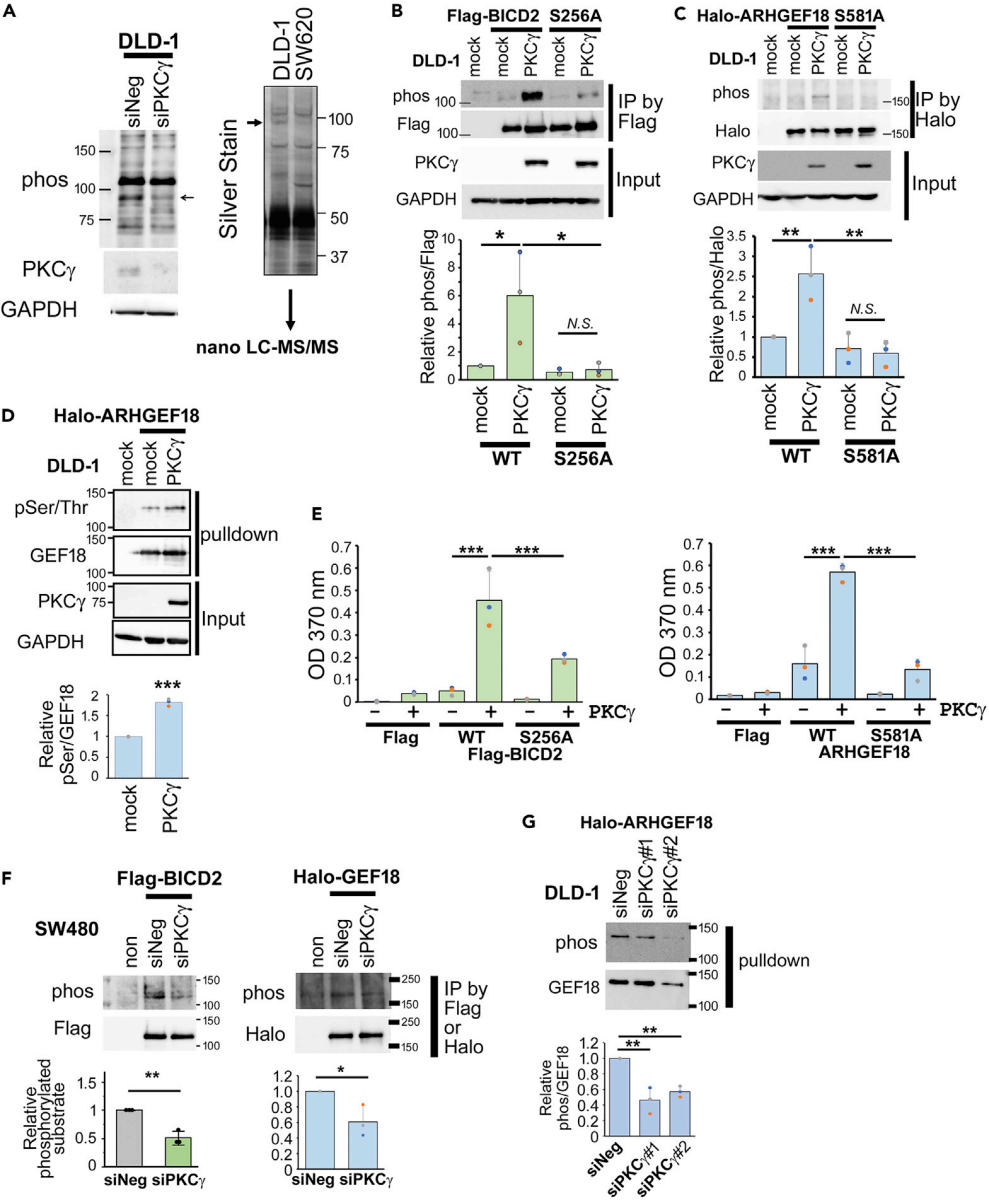
Identification of phosphorylation substrates of PKCγ (A) Lysates of DLD-1 cells transfected with siPKCγ or siNeg were analyzed by western blotting using an anti-phosphorylated PKC substrate antibody (phos) (left panel). Lysates of DLD-1 and SW620 cells were immunoprecipitated using an anti-phosphorylated PKC substrate antibody, separated by SDS-PAGE, and analyzed by silver staining (right panel). The indicated bands were analyzed using nano-LC–MS/MS. (B) FLAG-tagged BICD2 or (C) Halo-tagged ARHGEF18 was co-transfected with PKCγ in DLD-1 cells. Cell lysates were immunoprecipitated using anti-FLAG or Halo antibodies, and the phosphorylation levels were assessed using the anti-phosphorylated PKC substrate antibody (phos). The cell lysate before the commencement of immunoprecipitation served as the input (n = 3). (D) Halo-tagged ARHGEF18 and PKCγ was transfected in DLD-1, performed pull-down using Halo resin, treated with Halo TEV protease to elute ARHGEF18 (GEF18), and then assessed using anti-phosphorylated Ser/Thr antibody (pSer/Thr) and indicated antibodies (n = 3). (E) *In vitro* kinase assays were performed using purified PKCγ and substrate proteins as indicated (n = 3). (F) FLAG-tagged BICD2 or Halo-tagged ARHGEF18 was co-transfected with control siRNA (siNeg) or siPKCγ. Cell lysates were immunoprecipitated using anti-FLAG or Halo antibodies, and then the phosphorylation levels were assessed using the anti-phosphorylated PKC substrate antibody (phos) (n = 3). (G) Halo-tagged ARHGEF18 and siRNA were transfected in DLD-1 cells, followed by a pull-down using Halo resin, and then treated with Halo TEV protease to elute ARHGEF18 (GEF18). Assessment using the anti-phosphorylated PKC substrate antibody (phos) (n = 3) was then performed. (B–G) Data represent means ± SD. Statistical analysis was performed using Tukey’s multiple comparison of means test (B, C, E, and G) or Student’s *t* test (D and F). ∗p < 0.05; ∗∗p < 0.01; ∗∗∗p < 0.001. See also Table S3.

To identify phosphorylation substrate proteins for PKCγ, we explored the phosphorylated PKC substrates in DLD-1 using an anti-phosphorylated PKC substrate antibody. Western blot analysis revealed several candidate fractions, which are decreased by PKCγ knockdown (Figure 5A). The immunoprecipitation of phosphorylated PKC substrate proteins using an anti-phosphorylated PKC substrate antibody from both DLD-1 and SW620 also revealed the candidate fraction which is observed more intensely in DLD-1 than in SW620 (Figure 5A). Subsequent nano-liquid chromatography (LC)–mass spectrometry (MS)/MS of the fraction identified the candidate proteins (Table S3). Among them, we assessed proteins with a putative PKC recognition motif (serine residues surrounded by arginine [Arg] or lysine [Lys] at the −2 and +2 positions and a hydrophobic residue at the +1 position),^22^ whether they are phosphorylated by PKCγ. Because the candidate fraction identified in SDS-PAGE (Figure 5A) could be degradation products, we did not take the molecular weight into account. When Rho/Rac guanine nucleotide exchange factor 18 (ARHGEF18) or BICD cargo adaptor 2 (BICD2) were co-overexpressed with PKCγ in DLD-1 cells, the phosphorylation of ARHGEF18 or BICD2 was enhanced (Figures 5B–5D). To determine whether these proteins can be directly phosphorylated by PKCγ, we performed *in vitro* kinase assays using the isolated proteins. These revealed that ARHGEF18 and BICD2 can be directly phosphorylated by PKCγ (Figure 5E).

Consensus phosphorylation site motifs for PKCs typically contain basic amino acids ([Arg] or [Lys]) at positions −2 and +2 from the phosphorylation sites (serine [Ser] or threonine [Thr]), although this is not absolute.^22^ To determine the possible phosphorylation sites of the identified substrates, the Ser residues in the motifs were mutated to Ala, and the phosphorylation by PKC was assessed. When Ser-256 of BICD2 or Ser-581 of ARHGEF18 was mutated to Ala, the phosphorylation of these substrates by PKC was reduced in cells and *in vitro* (Figures 5B, 5C, and 5E). These results indicate that these Ser residues are the phosphorylation sites for PKCγ or affect its phosphorylation.

To elucidate whether these proteins are phosphorylated by endogenous PKCγ, we knocked down PKCγ in SW480 cells, and the phosphorylation of the relevant substrates was evaluated. The phosphorylation of BICD2 and ARHGEF18 was diminished by PKCγ knockdown (Figure 5F). In DLD-1 cells, phosphorylation of ARHGEF18 also decreased upon the knockdown of PKCγ (Figure 5G).

### PKCγ stabilizes ARHGEF18 protein, which maintains epithelial morphogenesis

**Figure 6:**
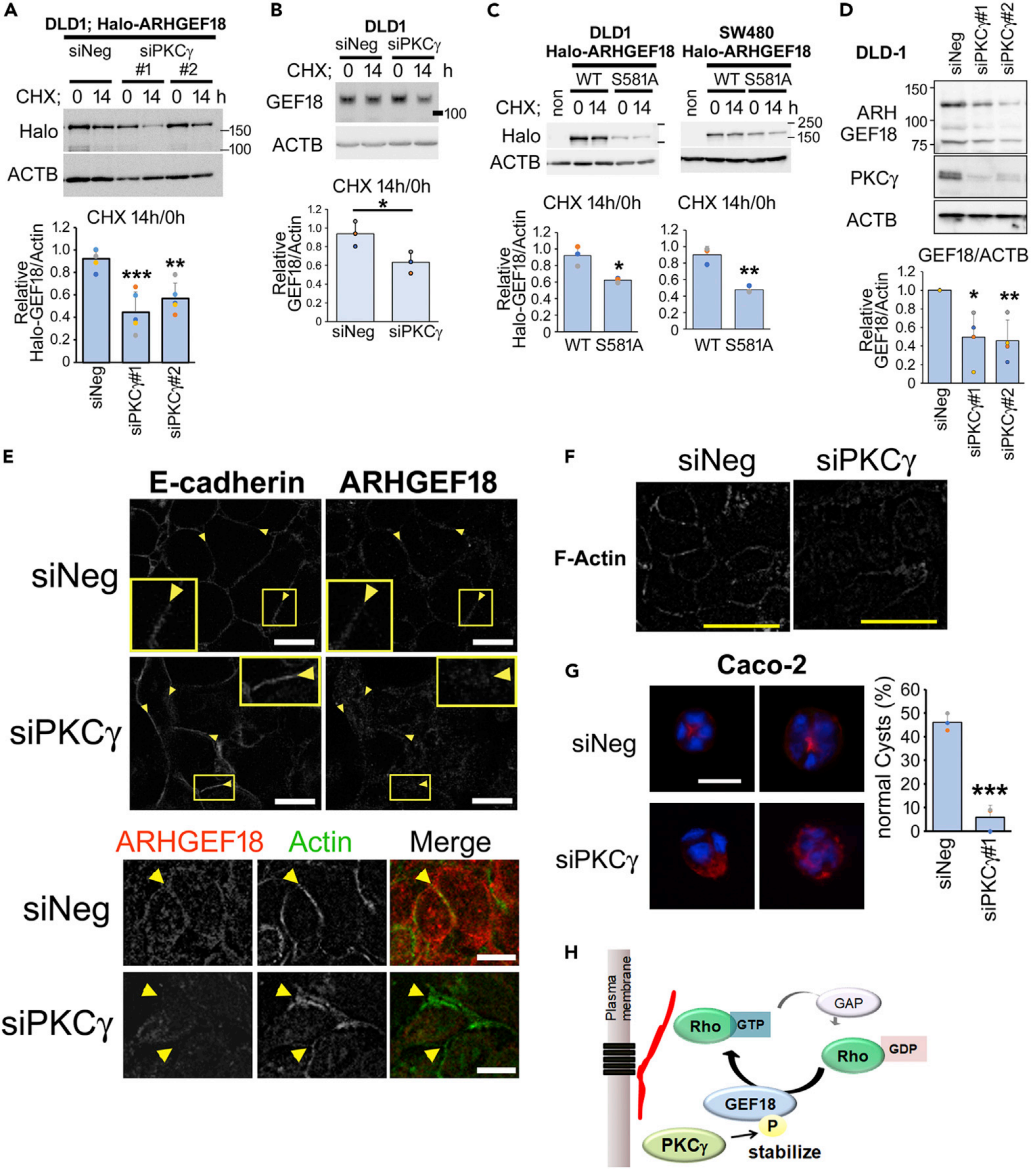
PKCγ stabilizes ARHGEF18 and maintains epithelial integrity (A) DLD-1 cells were transfected with Halo-ARHGEF18 and the indicated siRNAs. After 3 days, the cells were treated with cycloheximide (200 μg/mL) and then harvested at the indicated time points. The relative levels of Halo-ARHGEF18 normalized to ACTB were analyzed using western blotting (n = 3). (B) DLD-1 cells were transfected with indicated siRNAs. After 3 days, the cells were treated with cycloheximide (200 μg/mL) and then harvested at the indicated time points. The relative levels of ARHGEF18 normalized to ACTB were analyzed using western blotting (n = 3). (C) DLD-1 or SW480 cells were transfected with Halo-ARHGEF18 (WT or S581A), following which the cells were treated with cycloheximide (200 μg/mL) and harvested at the indicated time points. The relative levels of Halo-ARHGEF18 normalized to ACTB were analyzed using western blotting (n = 3). (D) DLD-1 cells were transfected with the indicated siRNAs. After 4 days, endogenous ARHGEF18 was detected using western blotting (n = 4). (E) DLD-1 cells were transfected with the indicated siRNAs. After 5 days, the cells were fixed with methanol and subjected to immunofluorescence staining with E-cadherin and ARHGEF18 antibodies (upper panels) or F-actin (Acti-stain 488 phalloidin; green) and ARHGEF18 antibodies (red) (lower panels). Scale bar = 10 μm. (F) DLD-1 cells were transfected with the indicated siRNAs. After 6 days, the cells were fixed with methanol and subjected to immunofluorescence staining with F-actin. Scale bar = 50 μm. (G) Caco-2 cells were transfected with the indicated siRNA, mounted in Matrigel, and then examined using immunostaining with Hoechst (blue) and phalloidin (red). The percentage of normally polarized cysts per total cell cluster containing 3–10 cells is shown in the bar graph (more than 85 clusters were counted from three experimental replicates). Scale bar = 50 μm. (H) A putative mechanism of epithelial maintenance by PKCγ. PKCγ phosphorylates and stabilizes ARHGEF18, which activates Rho A at cell-cell junctions to maintain epithelial polarity. (A–D and G) Data are presented as means ± SD. Statistical analysis was performed using Dunnett’s multiple comparison of means test (A and D) or Student’s *t* test (B, C, and G). ∗p < 0.05; ∗∗p < 0.01; ∗∗∗p < 0.001. See also Figure S4.

We observed a significant reduction in the amount of Halo-ARHGEF18 upon PKCγ knockdown (Figure S4A). This prompted us to examine whether PKCγ enhances the stability of ARHGEF18. The rate of ARHGEF18 degradation was measured in DLD-1 cells following treatment with cycloheximide, a protein synthesis inhibitor. Upon the siRNA-mediated suppression of PKCγ, the Halo-ARHGEF18 degradation rate increased compared to that in cells transfected with a control siRNA (Figure 6A). Endogenous ARHGEF18 also degraded faster when PKCγ was knocked down (Figure 6B). In contrast, the knockdown of PKCα did not significantly affect ARHGEF18 degradation (Figure S4B). Furthermore, the degradation rate of ARHGEF18 (S581A) was greater than that of the wild type (Figure 6C), suggesting the significance of ARHGEF18 phosphorylation by PKCγ in its stability. When PKCγ expression was suppressed, endogenous ARHGEF18 was also reduced (Figure 6D). Because ARHGEF18 is a guanine nucleotide exchange factor for RhoA, which activates RhoA at cell-cell junctions and promotes cell-cell junction assembly,^23^ we next assessed ARHGEF18 localization in DLD-1 cells. In control cells, ARHGEF18 localization at the cell-cell junction close to E-cadherin and apical actin filaments was observed (Figure 6E). In cells transfected with PKCγ-siRNA, E-cadherin and actin remained partially localized at the junction at day five post-transfection, but ARHGEF18 was diffusely distributed in the cells (Figure 6E). ARHGEF18 knockdown in CRC cells results in aberrant appearance of the junctional staining of peri-junctional F-actin, which is required for normal junction formation.^23^ F-actin staining away from cell junctions and redistribution of F-actin throughout the cells in PKCγ knockdown cells was also observed (Figure 6F). Because ARHGEF18 contributes to the maintenance of epithelial morphogenesis, such as 3D cyst formation observed in Caco-2 cells,^23^ we next assessed cyst formation. Caco-2 cells cultured in Matrigel form cysts with apical constricted actin.^24^ Although Caco-2 cells transfected with negative control siRNA formed normally polarized cysts with apically constricted actin, Caco-2 cells transfected with siRNA for PKCγ barely formed normal cysts (Figure 6G). These results suggest that PLCγ phosphorylates and stabilizes ARHGEF18 and regulates epithelial morphogenesis in CRC (Figure 6H).

## Discussion

To date, the role of PKCγ has not been well characterized in the field of cancer, although the role of PKCγ in neuronal diseases is well documented.^25^ Among them, SCA14, which is a type of spinocerebellar ataxia (a group of cerebellar diseases characterized by progressive ataxia and cerebellar atrophy), is caused by missense mutations in *PRKCG*. PKCγ dysregulation leads to the abnormal dendritic development of Purkinje cells, which induces the onset of SCA14.^25^ Because *PRKCG* expression in intestinal epithelium is much lower than that in neuronal tissues, the role of PKCγ in colorectal cancer progression is not well documented. In several human cancers, most PKC mutations result in their loss of function. Correction of a heterozygous PKCβ mutation via genome editing suppressed anchorage-independent growth and reduced CRC growth in a xenograft model, indicating that PKCβ has a tumor suppressive function.^14^ PKCα-knockout APC^Min/+^ mice model display an elevated risk of intestinal tumors,^13^ suggesting that PKCα suppresses colon cancer development, whereas multiple studies revealed that PKCα confers drug resistance to human CRC cells.^26^^,^^27^ In human CRC tissues, PKCα and PKCβ expressions were downregulated compared with the normal colonic epithelium.^28^ Moreover, disease progression is not associated with the protein levels of PKCβII, and there is no reduction in the disease–free survival time associated with low PKCβII expression in colorectal cancer epithelial tissue.^29^

In this study, we explored the role of PKCγ in several CRC cell lines harboring no *PRKCG* mutation.^30^ We clarified that PKCγ expression is lower in CRC tissues, especially in dedifferentiated CRC (Figure 2). PKCγ knockdown reduces E-cadherin expression and enhances malignant phenotypes in several CRC cell lines (Figures 3 and 4). As a possible mechanism of PKCγ-mediated E-cadherin regulation, we suggested ARHGEF18 modulation by PKCγ. PKCγ phosphorylates and stabilizes ARHGEF18 (Figures 5 and 6), which maintains junctional assembly and epithelial morphogenesis via RhoA activation.^23^ Because cell–cell junctions maintain E-cadherin expression and suppress EMT progression in CRC cells,^5^ downregulation of PKCγ might result in E-cadherin reduction because of junctional disassembly. In DLD-1 cells, vimentin expression was not induced by PKCγ knockdown (data not shown), while fibronectin expression was markedly induced (Figure 3G); we thus speculated that PKCγ knockdown initially induces partial reduction of epithelial traits and partial induction of mesenchymal traits, which also enhances malignant properties.

Several LOF PKC mutations act in a dominant-negative manner. This means that they decrease global endogenous PKC activity, which could be a sign of the interrelationships between the different isoforms of PKCs.^14^ Since PKCα is expressed in the colon epithelium and has a suppressive function in colon cancer development,^13^ we also assessed whether knockdown of PKCα affects E-cadherin expression. Knockdown of PKCα in CRC cells did not result in a reduction in E-cadherin level (Figure S3), suggesting a functional difference between PKCα and PKCγ. The precise functional comparison of cPKCs remains to be fully resolved.

### Limitations of the study

In this study, we did not rescue the PKCγ knockdown phenotype by ARHGEF18 overexpression, because it is speculated that many other phosphorylation substrates for PKCγ might regulate cellular phenotype coordinately. In that situation, the importance of ARHGEF18 modulation by PKCγ remains obscure. Further investigations are required to identify many other substrate proteins for PKCγ and to elucidate the importance of their phosphorylation by PKCγ. Appropriate regulation of PKCγ activity at the cell-cell junction may also be a prerequisite for its tumor suppressive effect and the role of PKCγ in other cellular components may be different. Further analysis of PKCγ with respect to other identified binding proteins is required.

## STAR★Methods

### Key resources table

**Table:** 

REAGENT or RESOURCE	SOURCE	IDENTIFIER
**Antibodies**		

Mouse monoclonal anti-E-cadherin	BD Biosciences	Cat#610182, RRID:AB_397581
Rabbit polyclonal anti-Flag	Sigma	Cat#F7425, RRID:AB_439687
Mouse monoclonal anti-Flag	Sigma	Cat#F1804, RRID:AB_262044
Rabbit polyclonal Fibronectin	Merck	Cat#Ab1954, RRID: AB_11213226
Mouse monoclonal anti-GAPDH	Santa Cruz	Cat#sc-32233, RRID:AB_627679
Mouse monoclonal anti-β-actin	Sigma	Cat#A5441, RRID:AB_476744
Mouse monoclonal anti-Halo	Promega	G921A
Rabbit polyclonal anti-Halo	Promega	G928A
Rabbit polyclonal anti-phospho Akt (Ser473)	Cell Signaling	Cat #9271, RRID:AB_329825
Rabbit polyclonal anti-phospho PKC substrate	Cell Signaling	Cat#2261, RRID:AB_330310
Mouse monoclonal anti-PKCα	BD Biosciences	Cat#610107, RRID: AB_397513
Mouse monoclonal anti-PKCβ	Santa Cruz	Cat#sc-13149, RRID: AB_628144
Mouse polyclonal anti-PKCγ for Figures 1A and 1B (SW480), Figures 2, 3, and 6	Abnova	Cat# H00005582-A01, RRID:AB_463355
Rabbit polyclonal anti-PKCγ for Figure 1B (DLD-1)	Santa Cruz	Cat#sc-211, RRID:AB_632234
Mouse monoclonal anti-PKCγ for Figures 5B–5D	Santa Cruz	Cat#sc-166385, RRID:AB_2018059
Mouse monoclonal anti-Vimentin	Santa Cruz	Cat#sc-32322, RRID:AB_628436
Goat anti-ARHGEF18	Everest BIOTECH	Cat#EB06163, RRID:AB_2227541

**Chemicals, peptides, and recombinant proteins**		

Bryostatin 1	Sigma	B7431
Cycloheximide	Sigma	C4859
Oxaliplatin	Wako	156–02691
Puromycin	Sigma	P8833

**Critical commercial assays**		

Dual-luciferase Reporter Assay System	Promega	E1910
TMB Super Sensitive HRP Substrate	Surmodics	TMBS-0100-01

**Deposited data**		

Mendeley dataset	https://data.mendeley.com/datasets/yzp4k8frj4/draft?a=c8f44734-9ace-43b6-8860-2e7f1efdcb12	N/A

**Experimental models: Cell lines**		

CaCo-2	RIKEN BRC	RCB0988, RRID:CVCL_0025
SW620	ATCC	Cat#CCL-227, RRID:CVCL_0547
SW480	ATCC	Cat#CCL-228, RRID:CVCL_0546
DLD-1	JCRB Cell Bank	Cat#JCRB9094, RRID:CVCL_0248
WiDr	ATCC	Cat#CCL-218, RRID:CVCL_2760
HEK293	JCRB Cell Bank	Cat#JCRB9068, RRID:CVCL_0045
shPKCγ expressing DLD-1	This paper	N/A

**Oligonucleotides**		

Allstars Negative Control siRNA	Qiagen	SI03650318
si PKCγ-#1	Qiagen	SI04997174
si PKCγ-#2	Qiagen	SI03078306
si PKCα	Qiagen	SI04997174
human *PRKCG*-Forward; GTTTAAGGAGCCCCATGCAG	FASMAC	N/A
human *PRKCG*-Reverse; CCCTCAGCATCCAGCATCAC	FASMAC	N/A
human *ACTB*-Forward; GCCCTGGCACCCAGCACAAT	FASMAC	N/A
human *ACTB*-Reverse; GGAGGGGCCGGACTCGTCAT	FASMAC	N/A
human *GAPDH*-Forward; AGCCTCCCGCTTCGCTCTCT	FASMAC	N/A
human *GAPDH*-Reverse; CCAGGCGCCCAATACGACCA	FASMAC	N/A
human *SNAI1*-Forward; CTGCGGGAAGGCCTTCTCT	FASMAC	N/A
human *SNAI1*-Reverse; CGCCTGGCACTGGTACTTCTT	FASMAC	N/A

**Recombinant DNA**		

pMXs-IN-Snail *(SNAI1*)	This paper	N/A
pcDNA3.1- PKCγ (*PRKCG*)	This paper	N/A
pcDNA3.1- PKCα (*PKRCA*)	This paper	N/A
pFN21A-Halo-*PRKCB*	Promega	FHC10533
pcDNA3.1- Flag-*BICD2*	This paper	N/A
pFN21A-Halo-*ARHGEF18*	Promega	FHC01977
pSUPER retro puro- shPKCγ-#1 targeting; 5′GGCCATCATGGAACAAACTGT-3′	This paper	N/A
pSUPER retro puro- shPKCγ-#2 targeting; 5′GCGAGAACTTTGACAAGTTCT -3′	This paper	N/A
pGL4.10-luc2 vector	Promega	E665A
pGL4.74-hRluc vector	Promega	E692A
pGL4.10-luc2-pPKCγ-Luc	This paper	N/A

**Software and algorithms**		

R (v4.0.3)	http://www.r-project.org/	N/A
CS Analyzer4 (Atto)	https://www.atto.co.jp/site/products/geldocumentation/Image-analysis-software/Image-Analysis-Software2	N/A

**Deposited data**		
Raw data for figures	https://data.mendeley.com/datasets/yzp4k8frj4/draft?a=c8f44734-9ace-43b6-8860-2e7f1efdcb12	N/A

### Resource availability

#### Lead contact

Further information and requests for resources and reagents should be directed to and will be fulfilled by the lead contact, Reiko Satow (rsatow@toyaku.ac.jp).

#### Materials availability

The plasmids generated in this study are available from the lead contact on reasonable request.

### Experimental model and subject details

#### Cell culture and reagents

The colorectal adenocarcinoma cell lines SW620, SW480 and WiDr were obtained from the American Type Culture Collection (Manassas, VA, USA). DLD-1 and HEK293 cell lines were obtained from JCRB Cell Bank (National Institute of Health Sciences, Tokyo, Japan). Caco-2 cells were obtained from the RIKEN Bioresource Center (RIKEN BRC) (Ibaraki, Japan). These cell lines were re-validated by short tandem repeat profiling in 2016 (Promega, Madison, WI, USA). The cells were maintained as described previously.^31^ Bryostatin 1 and cycloheximide were obtained from Sigma (B7431, C4859) and oxaliplatin was obtained from Wako (156-02691).

#### Animal experiments

Five million DLD-1 cells stably expressing shPKCγ were suspended in 0.1 mL of PBS (PBS) and subcutaneously inoculated into the flanks of 5-week-old female BALB/c nu/nu nude mice (CLEA, Tokyo, Japan). Animal experiments were approved by the institutional ethics committee and performed in compliance with the guidelines for Laboratory Animal Research of the Tokyo University of Pharmacy and Life Sciences (Tokyo, Japan).

### Method details

#### Western blot analysis

Whole-cell lysate was collected using Laemmli sample buffer. Western blot analysis was performed as described previously,^31^ with some modifications. Primary antibodies targeting E-cadherin (610182, BD Biosciences, San Jose, CA, USA), FLAG tag (F7425, Sigma), Fibronectin (AB1954, Merck), glyceraldehyde 3-phosphate dehydrogenase (GAPDH) (sc32233, Santa Cruz, Dallas, TX, USA), β-actin (A5441, Sigma), Halo tag (G921A, Promega), phospho PKC substrate (#2261, Cell Signaling), phospho Akt (Ser473) (#9271, Cell Signaling), PKCγ (sc211 (for Figure 1B; DLD-1) or sc166385 (for Figures 5B–5D), Santa Cruz or H00005582, Abnova (for Figures 1A and 1B (SW480), Figures 2, 3, and 6)), vimentin (sc32322, Santa Cruz) and ARHGEF18 (EB06163, Everest Biotech) were used. Images were obtained using ImageQuant TL (GE Healthcare, Piscataway, NJ, USA) or LuminoGraph I (Atto) and quantified using CS Analyzer4 (Atto).

#### Plasmids, siRNA, and transfection

The open reading frame (ORF) of human *SNAI1* (Gene symbol; SNAI1) was amplified by PCR and subcloned into the pMXs-IN vector. The ORFs of human *PRKCA*, *PRKCG* and *BICD2* were amplified and subcloned into pcDNA3.1 (Invitrogen, Carlsbad, CA, USA). We have confirmed that none of these sequences contain mutations. Halo-tagged *ARHGEF18* and *PRKCB* (PKCβ) were obtained from Promega (FHC01977 and FHC10533, respectively). The pSUPER retro puro retroviral vector (Oligoengine) was used for short hairpin RNA (shRNA) expression with the following targeting sequences: shPKCγ-#1, 5′- GGCCATCATGGAACAAACTGT -3′; and shPKCγ-#2, 5′- GCGAGAACTTTGACAAGTTCT -3′. To obtain stable cell lines, transfected cells were selected with 1 μg/mL puromycin (P8833, Sigma) or 1,000 μg/mL G418 (10131035, Thermo) and the resulting single clones were analyzed for stable expression.

Negative control siRNA (siNeg) and siRNAs for *PKC*γ (Qiagen, Hilden, Germany) were used at final concentrations of 20 nM. Transient transfections were performed using polyethylenimine, Lipofectamine 2000, or Lipofectamine RNAi max (Invitrogen), according to the manufacturer’s protocols. In every experiment, the total amount of transfected DNA or siRNA was adjusted using the relevant empty vector or negative control siRNA, respectively.

#### Luciferase assay

The luciferase reporter construct pPKCγ-Luc was constructed by amplifying the *PRKCG* proximal promoter region using the primers pPKCγ-F (5′- CAC AAG ATC TGA GAT TGG GTC AGA GAG AAA GGGA -3′) and pPKCγ-R (5′- ACC AGC CAT GGC CCC AGAA -3′), and subcloned into the *Bgl*II–*Nco*I region of the pGL4.10-luc2 vector (Promega). pPKCγ-Luc mutants were constructed using the following primers: m1-F, 5′- ATC CTG TTT CCC CCA AGA AAG GCA -3′; m2-F, 5′- ATC CTG GAG GTG CCT TGC CCC T -3′; m3-F, 5′- ATG GTG CCG GAG CTG GAG CTC -3′; m4-F, 5′- GGG AGG AAT TTT GTC CCG TGT CTC CGGG -3′; and m5-F, 5′- ATC GTG TGG GGG GCG GGGA -3′. Cells were co-transfected with the pPKCγ luciferase reporter constructs and a pGL4.70 internal control plasmid (Promega) using Lipofectamine 2000 reagent (Invitrogen). The TOP/FOP FLASH assay was performed to evaluate T-cell factor/lymphocyte enhancer factor transcriptional activity. Luciferase activity was then measured using the Dual-luciferase Reporter Assay System (Promega), as described previously.^11^^,^^32^

#### Immunohistochemistry

Human colon carcinoma tissue arrays with matched adjacent normal colon tissue were purchased from US Biomax (Rockville, MD, USA). The immunostaining was performed as described previously^11^ with anti-PKCγ antibody (H00005582, Abnova).

#### Immunocytochemistry

Immunofluorescence microscopy was performed as described previously^11^ using antibodies targeting E-cadherin (580061, BD Biosciences) and ARHGEF18 (EB06163, Everest Biotech). F-actin was stained with Acti-stain 488 phalloidin (Cytoskeleton Inc.). Images were obtained using a BZ-X700 microscope (Keyence) with sectioning and z stack modules.

#### RNA isolation, cDNA synthesis, and quantitative real-time PCR (qRT-PCR)

RNA isolation, cDNA synthesis, and qRT-PCR were performed as described previously.^11^ To calculate the relative mRNA expression in the qRT-PCR analysis, standard curve lines for each primer set were plotted and then used for analysis. The primers used were *PRKCG* (PKCγ), F; GTTTAAGGAGCCCCATGCAG, R; CCCTCAGCATCCAGCATCAC, *ACTB*, F; GCCCTGGCACCCAGCACAAT, R; GGAGGGGCCGGACTCGTCAT, *GAPDH*, F; AGCCTCCCGCTTCGCTCTCT, R; CCAGGCGCCCAATACGACCA, *SNAI1*, F; CTGCGGGAAGGCCTTCTCT R; CGCCTGGCACTGGTACTTCTT.

#### Cell proliferation and scratch migration assays

Cell proliferation were assessed as described previously.^11^ For scratch migration assay, cells were seeded in 96-well plate at a density of 0.25 × 10^5^ cells per well. After 6 days, cells were starved for 24 h and the cell layers were scratched with sterile pipette tip from top to bottom. Images at the same position were obtained using InCell Analyzer 2000 (GE Healthcare) before and after 19 h incubation.

#### Soft agar colony formation assay

The cells were layered (10^3^ cells/well) in base agar (0.8%)-coated 6-well plates with room temperature agar (0.5%) in RPMI 1640 medium. The plates were then incubated at 37°C for 2–3 weeks, the number of colonies was counted, and images were obtained.

#### Immunoprecipitation

Cells were lysed in immunoprecipitation buffer (150 mM NaCl, 50 mM Tris-HCl at pH 7.5, 0.2% deoxycholate, and 0.5% Nonidet P-40) supplemented with a phosphatase inhibitor cocktail (Sigma-Aldrich) and a protease inhibitor cocktail (Roche, Mannheim, Germany). The lysate was incubated with anti-FLAG M2 monoclonal antibody (F1804, Sigma) or anti-Halo antibody (G928A, Promega) at 4°C overnight and then precipitated using protein G (GE Healthcare). Immunoprecipitates were washed four times with ice-cold PBS and resolved by sodium dodecyl sulfate-polyacrylamide gel electrophoresis.

#### Silver staining and nano-LC–MS/MS

Silver staining was performed using a Silver Stain kit for MS (Apro Science, Tokushima, Japan), according to the manufacturer’s protocol. The tryptic peptides were fractionated using an UltiMate 3000 RSLCnano system (Thermo Fisher Scientific) and analyzed using an Orbitrap Elite (Thermo Fisher Scientific). Tandem mass spectra were extracted using Proteome Discoverer version 1.4. All MS/MS samples were analyzed using Mascot (Matrix Science, London, UK; version 2.5.1), which was set up to search the NCBIprot_Homo_sapiens_20180308 database (1,228,116 entries), assuming the use of the digestion enzyme trypsin (Support Center for Advanced Medical Sciences, Tokushima University Graduate School of Biomedical Sciences).

#### Recombinant protein purification

HEK293 cells were transfected with FLAG or Halo-tagged expression vectors. After 2–3 days, FLAG-tagged protein-expressing cells were lysed in immunoprecipitation buffer (150 mM NaCl, 50 mM Tris-HCl at pH 7.5, 0.2% deoxycholate, and 0.5% Nonidet P-40) supplemented with a protease inhibitor cocktail (Roche, Mannheim, Germany); anti-FLAG affinity agarose gel (A2220, Sigma) was added and the mixture was incubated overnight at 4°C. The gel was washed thoroughly with PBS and FLAG-tagged protein was eluted by adding FLAG peptide. The Halo-tagged protein was purified using HaloLink resin (G1914, Promega), according to the manufacturer’s protocol.

#### *In vitro* kinase assay

Purified substrate proteins (0.3–1 μg/mL) were loaded into ELISA plates (Iwaki) at 4°C overnight. After washing twice with Tris-buffered saline supplemented with 0.05% Tween 20 (TTBS), 50 μL of kinase buffer (150 mM NaCl, 20 mM HEPES at pH 8, 10 mM MgCl_2_, 1 mM DTT, and 20 μM ATP, 0.5 μM or 1 mM CaCl_2_) with 1 μM bryostatin 1 and 1 μg/mL PKC was added and the plate incubated at 30°C for 1 h. After washing twice with TTBS, anti-phosphorylated PKC substrate (1/1000) was added and the plate was incubated for 1 h; then, HRP-conjugated anti-rabbit IgG (½,000) was added for 30 min, the plate was washed three times with TTBS, incubated with 50 μL of TMB Super Sensitive HRP Substrate (Surmodics) for 30 min, and the absorbance was read at *370 nm* using a microplate reader.

### Quantification and statistical analysis

#### Statistical analysis

Data were analyzed using the R statistical software package (version 4.0.3). The statistical analyses are described in the Figure legends. The tests used included Wilcoxon signed-rank test, Fisher exact test, two-tailed unpaired Mann–Whitney U-test, and two-tailed unpaired Student’s *t* test. Dunnett’s or Tukey’s test was used for post-hoc multiple comparisons. *p* < 0.05 was considered to represent statistical significance (∗p < 0.05; ∗∗p < 0.01; ∗∗∗p < 0.001). Data are presented as the mean ± standard deviation (s.d.) (n ≥ 3 independent experimental replicates), unless otherwise stated.

#### Analysis of gene expression datasets

Using gene expression datasets (GSE39582) from human cancers that included information on survival, the patients were divided into high and low PKC expression groups. A Kaplan–Meier survival curve was constructed in Survfit (R package). p values were calculated using the log rank test in Survdiff (R package).

### Additional resources

Supplemental information contains four figures and three tables.

## Data Availability

The datasets used during the current study are available from the lead contact on reasonable request. Our dataset was also deposited as Mendeley dataset at [https://data.mendeley.com/datasets/yzp4k8frj4/draft?a=c8f44734-9ace-43b6-8860-2e7f1efdcb12].
