# Supplementary material for: Downregulation of protein kinase C gamma reduces epithelial property and enhances malignant phenotypes in colorectal cancer cells
Source: iScience. 2022 Nov 4;25(12):105501. doi: 10.1016/j.isci.2022.105501 (PMC9672935; doi:10.1016/j.isci.2022.105501)
Supplement: Document S1. Figures S1–S4 and Tables S1–S3 [file mmc1.pdf]

iScience, Volume *25*

Supplemental information

Downregulation of protein kinase C gamma reduces epithelial property and enhances malignant phenotypes in colorectal cancer cells

Reiko Satow, Yudai Suzuki, Shinobu Asada, Sae Ota, Masashi Idogawa, Shiori Kubota, Noi Ikeo, Atsuko Yoneda, and Kiyoko Fukami

SUPPLEMENTAL INFORMATION

Supplementary Information consists of four figures and three tables

Supplementary Figure 1. Specificity of PKC antibodies used in this study, Related to Figure 1, 2, 3, 5, 6, S3.

(A) HEK293 cells were transfected with the indicated plasmids. The cell lysates were assessed by western blotting using the indicated antibodies. (B) DLD-1 cells were transfected with siRNA for PKCγ and assessed by western blotting using an anti-PKCγ antibody (sc-211).

Supplementary Figure 2. SNAI1 represses PKCγ promoter activity, Related to Figure 1.

(A) Hela cells were transfected with pcDNA (empty vector) or *SNAI1*/pcDNA and the pPKCγ-Luc reporter vector. (B) Schematic of the proximal promoter region of *PRKCG* and the reporter construct (pPKCγ-Luc). Hela cells were transfected with pcDNA (empty vector; mock) or *SNAI1*/pcDNA and the pPKCγ-Luc reporter vector. (A, B) Luciferase activities were normalized to the activity of the internal control (*Renilla* luciferase) and the relative values are indicated ($n = 3$). Data represent means ± s.d.. Statistical analysis was performed using Student's *t*-test. *$p < 0.05$; **$p < 0.01$; ***$p < 0.001$.

Supplementary Figure 3. Knockdown of PKCγ, but not PKCα, reduces E-cadherin expression, Related to Figure 3.

Caco-2, WiDr, SW480, and DLD-1 cells were transfected with siRNA for PKCα, and

after 6 days, the cells were assessed by western blotting using the indicated antibodies.

Supplementary Figure 4. Knockdown of PKCγ, but not PKCα, reduces ARHGEF18 protein, Related to Figure 6.

(A) SW480 cells transfected with Halo-ARHGEF18 and the indicated siRNAs were analyzed using western blotting. The relative levels of Halo-ARHGEF18 normalized to GAPDH are shown (n = 3). (B) DLD-1 cells were transfected with Halo-ARHGEF18 and the indicated siRNAs. After 3 days, the cells were treated with cycloheximide (200 μg/mL) and then harvested at the indicated time points. The relative levels of Halo-ARHGEF18 normalized to ACTB were analyzed using western blotting (n = 3). Data are presented as means ± s.d.. Statistical analysis was performed using Dunnett's multiple comparison of means test (A) or Student's t-test (B). **$p < 0.01$.

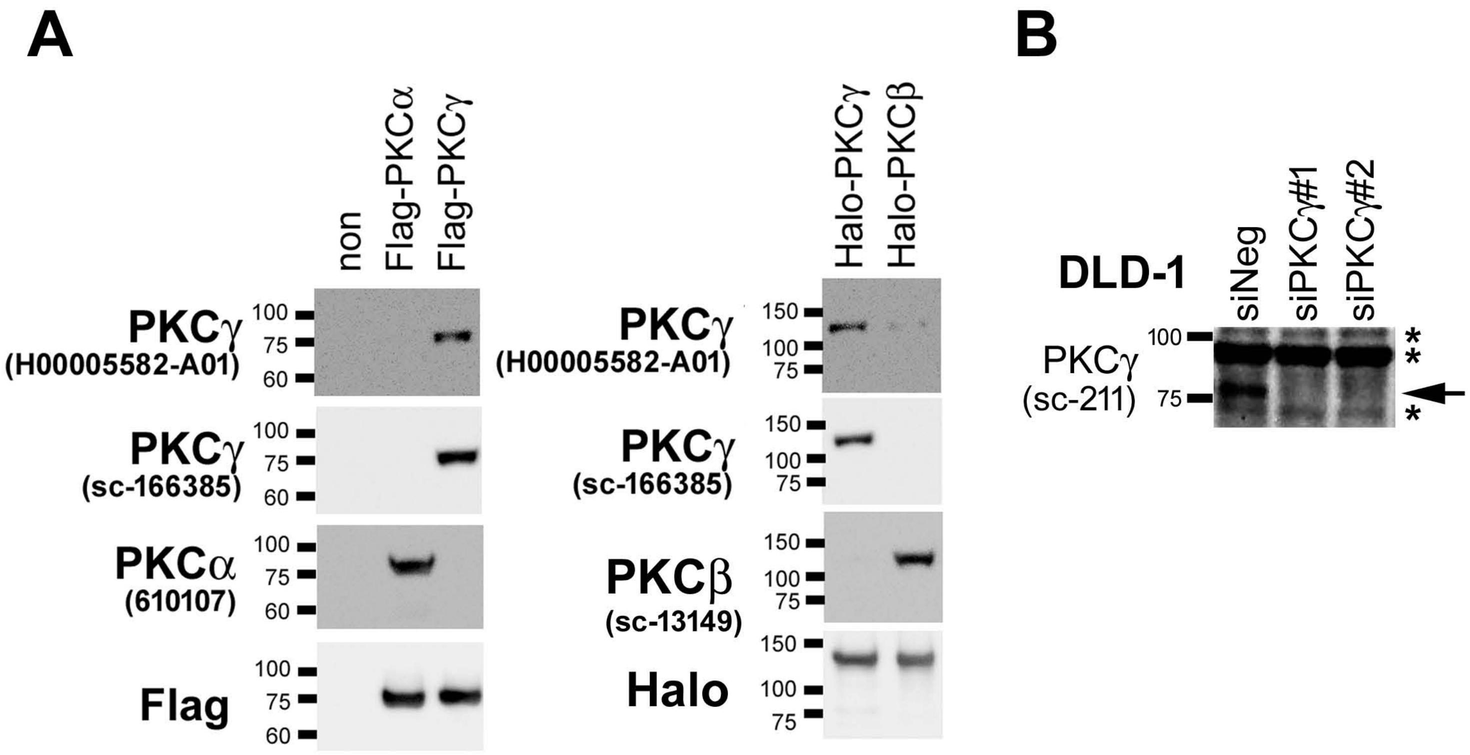

Supplementary Figure 1. Specificity of PKC antibodies used in this study, Related to Figure 1, 2, 3, 5, 6, S3

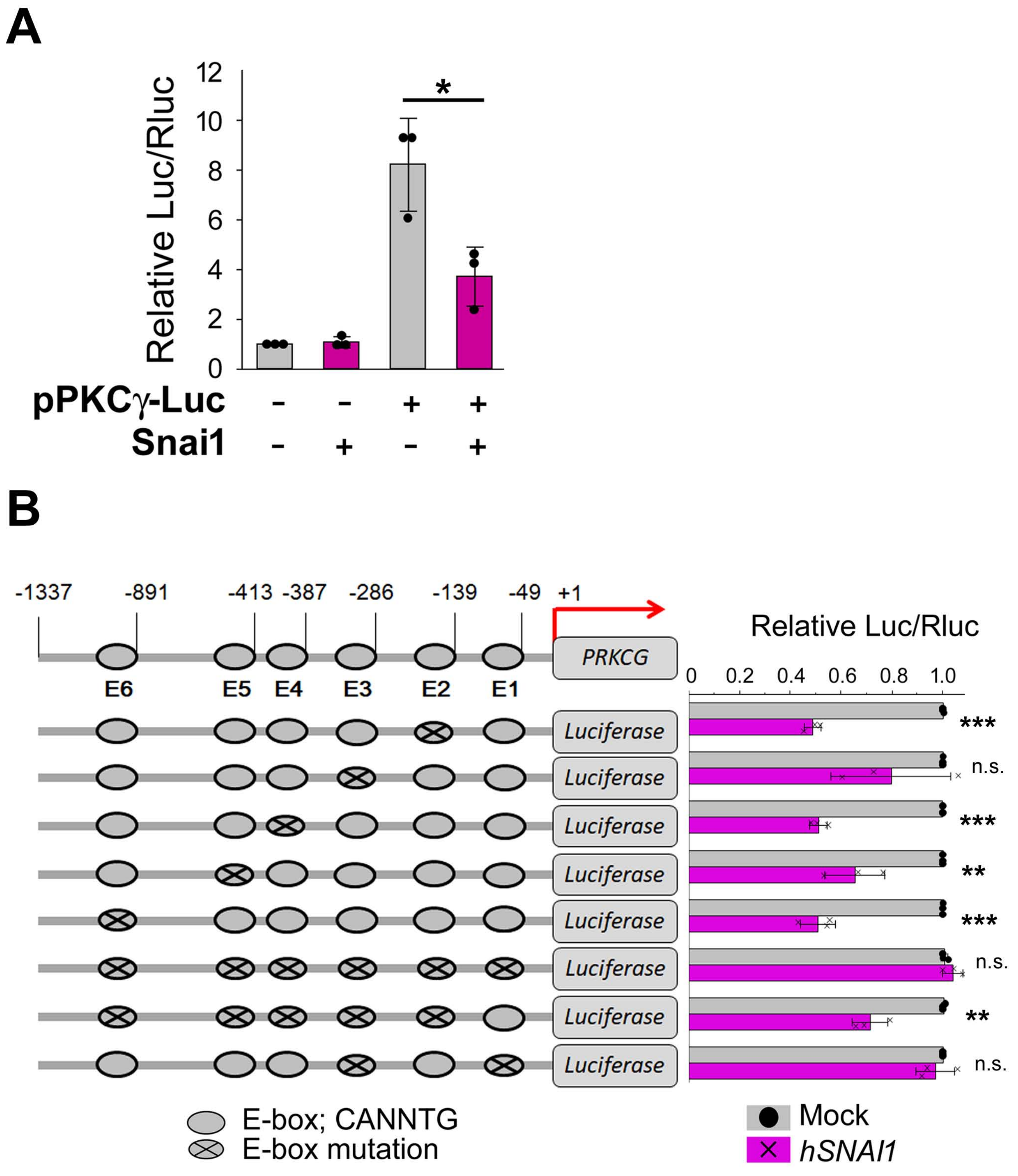

Supplementary Figure 2. SNAI1 represses PKCγ promoter activity, Related to Figure 1

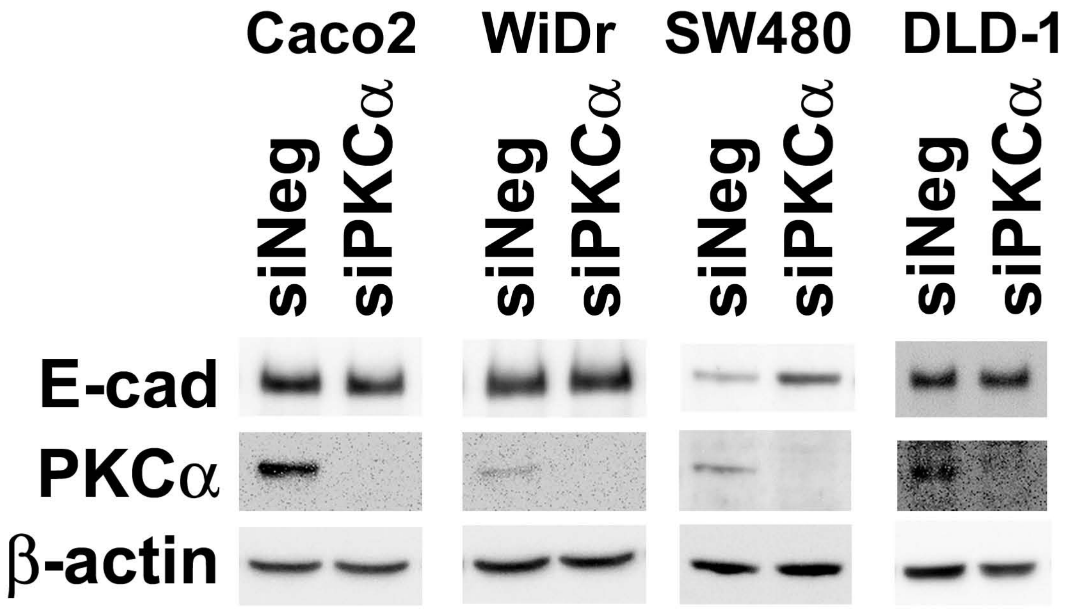

Supplementary Figure 3. Knockdown of PKCγ, but not PKCα reduces E-cadherin expression, Related to Figure 3

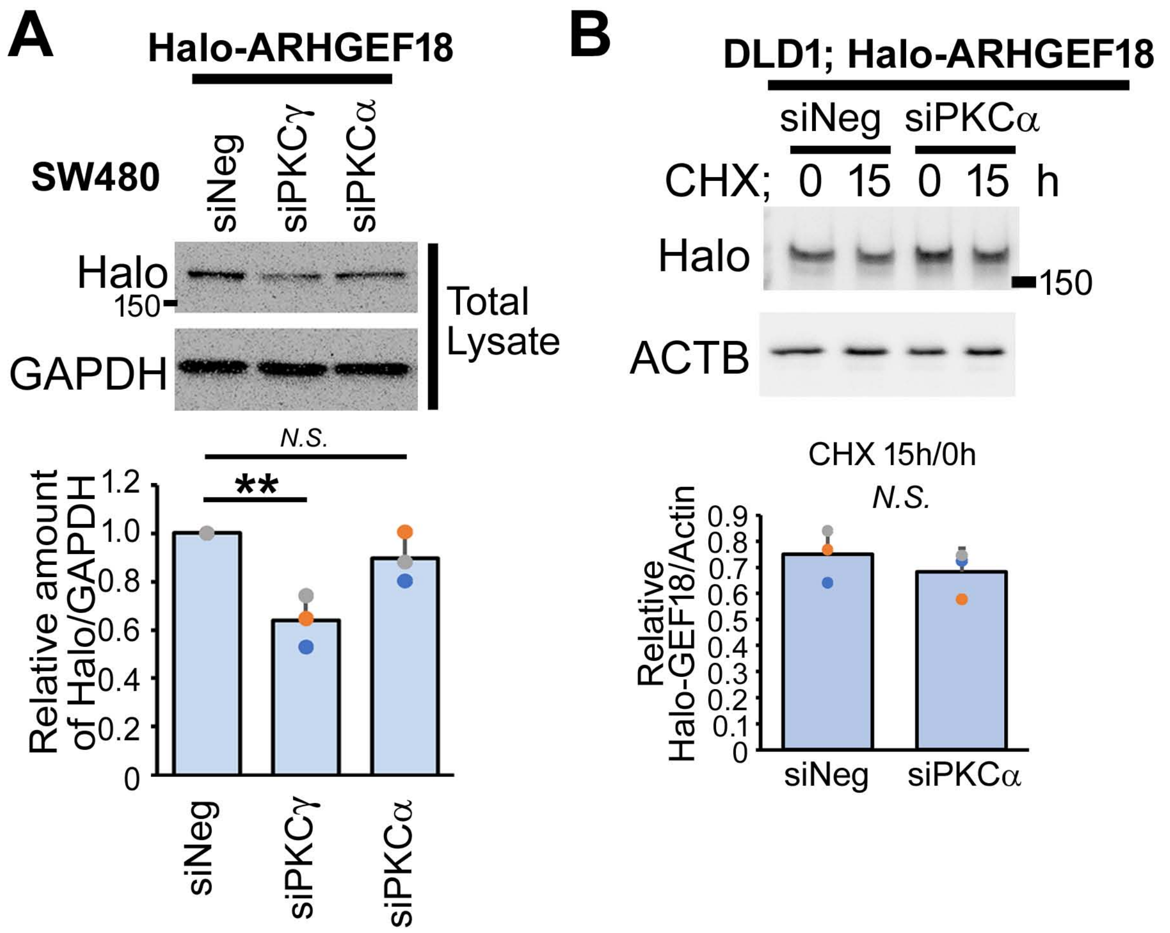

Supplementary Figure 4. Knockdown of PKCγ but not PKCα reduces ARHGEF18 protein, Related to Figure 6

Table S1

Features of the CRC cell lines used in this study, Related to Figure 1.

Cell line	PRKCG	KRAS	BRAF	TP53	Characteristics
Caco-2	wt	wt	wt	E204X	Established from an adenocarcinoma. Polarized and shows enterocytic differentiation[S1].
DLD-1	wt	G13D	wt	S241F	Established from a moderately to poorly differentiated adenocarcinoma. Epithelial[S2].
LoVo	wt	G13D;A14V	wt	wt	Established from a metastatic nodule in the left supraclavicular region. Cuboidal and columnar and rarely polygonal[S3].
HCT116	H456R, A378V	G13D	wt	wt	Established from colon. Closely packed polygonal cells[S4].
SW480	wt	G12V	wt	R273H;P309S	Primary adenocarcinoma from the same patient of SW620. Mixture of epithelial cells and individual bipolar cells, often exhibiting microvilli[S5].
SW620	wt	G12V	wt	R273H;P309S	Isolated from a lymph node metastasis. Dedifferentiated, no microvilli[S5].
WiDr	wt	wt	V600E	R273H	Established from a primary adenocarcinoma. Epithelial[S6].

Table S2

Relationships between the expression of PKCγ and the clinicopathologic features of colorectal cancer, Related to Figure 2. *P* values were determined using Fisher's exact test.

	**PKCγ Expression in Tumor Samples**				
**Variable**	0	1	2	3	*P*-value
**Sex**					**N. S.**
**Male**	14	6	4	1	
**Female**	13	5	7	1	
**Age (years)**					**N. S.**
**<53**	13	5	4	1	
**>=53**	14	6	7	1	
**Differentiation**					**0.001115**
**Well**	1	1	4	1	
**Moderate**	13	10	6	1	
**Poor**	13	0	1	0	
**Stage**					**0.2524**
**I**	0	1	0	0	
**II**	9	1	2	1	
**III**	11	8	8	1	
**IV**	7	1	1	0	

N.S. = not significant.

Table S3

A Mascot search revealed the candidate substrate proteins for PKCγ, Related to Figure 5. Of these, we interrogated the genes in bold, which have putative PKC recognition motifs.

**Sample 1**	
**Gene Symbol**	Total Count
CTNND1	36
MYO1C	32
ILF3	25
MCM6	23
**ARHGEF18**	21
GANAB	19
MCM4	18
CTNNA1	13
**BICD2**	13
DNM2	12
PRPF6	12
MYO1D	12
LIMA1	11
GTF3C3	11

REFERENCES

S1. Chantret, I., Barbat, A., Dussaulx, E., Brattain, M.G., and Zweibaum, A. (1936). Epithelial Polarity, Villin Expression, and Enterocytic Differentiation of Cultured Human Colon Carcinoma Cells: A Survey of Twenty Cell Lines1.

S2. Dexter, D.L., Spremulli, E.N., Fligiel, Z., Barbosa, J.A., Renee Vogel, B., and VanVOORHEES, A. (1981). Heterogeneity of Cancer Cells from a Single Human Colon Carcinoma.

S3. Drewinko, B., Romsdahl, M.M., Yang, L.Y., Ahearn, M.J., and Trujillo, J.M. (1976). Establishment of a human carcinoembryonic antigen-producing colon adenocarcinoma cell line. Cancer Res *36*, 467–475.

S4. Brattain, M.G., Fine, W.D., Khaled, F.M., Thompson, J., and Brattain, D.E. (1981). Heterogeneity of Malignant Cells from a Human Colonie Carcinoma1.

S5. Leibovitz, A., Stinson, J.C., McCombs, W.B., McCoy, C.E., Mazur, K.C., and Mabry, N.D. (1976). Classification of human colorectal adenocarcinoma cell lines. Cancer Res *36*, 4562–4569.

S6. Noguchi, P., Wallace, R., Johnson, J., Earley, E.M., O'Brien, S., Ferrone, S., Pellegrino, M.A., Milstien, J., Needy, C., Browne, W., et al. (1979). Characterization of WiDr: A human colon carcinoma cell line. In Vitro *15*, 401–408. 10.1007/BF02618407.
